# Involvement of Glutaredoxin and Thioredoxin Systems in the Nitrogen-Fixing Symbiosis between Legumes and Rhizobia

**DOI:** 10.3390/antiox7120182

**Published:** 2018-12-05

**Authors:** Geneviève Alloing, Karine Mandon, Eric Boncompagni, Françoise Montrichard, Pierre Frendo

**Affiliations:** 1 Université Côte d’Azur, INRA, CNRS, ISA, France; Genevieve.Alloing@unice.fr (G.A.); mandon@unice.fr (K.M.); Eric.BONCOMPAGNI@univ-cotedazur.fr (E.B.); 2 IRHS, INRA, AGROCAMPUS-Ouest, Université d’Angers, SFR 4207 QUASAV, 42 rue Georges Morel, 49071 Beaucouzé CEDEX, France; francoise.montrichard@univ-angers.fr

**Keywords:** thioredoxin, glutaredoxin, legume plant, symbiosis, redox homeostasis, stress

## Abstract

Leguminous plants can form a symbiotic relationship with Rhizobium bacteria, during which plants provide bacteria with carbohydrates and an environment appropriate to their metabolism, in return for fixed atmospheric nitrogen. The symbiotic interaction leads to the formation of a new organ, the root nodule, where a coordinated differentiation of plant cells and bacteria occurs. The establishment and functioning of nitrogen-fixing symbiosis involves a redox control important for both the plant-bacteria crosstalk and the regulation of nodule metabolism. In this review, we discuss the involvement of thioredoxin and glutaredoxin systems in the two symbiotic partners during symbiosis. The crucial role of glutathione in redox balance and S-metabolism is presented. We also highlight the specific role of some thioredoxin and glutaredoxin systems in bacterial differentiation. Transcriptomics data concerning genes encoding components and targets of thioredoxin and glutaredoxin systems in connection with the developmental step of the nodule are also considered in the model system *Medicago truncatula*–*Sinorhizobium meliloti*.

## 1. Introduction

Most terrestrial plants establish symbiotic relationships with fungi or bacteria that provide nutrients for their growth [1,2]. Nitrogen and phosphorous are critical determinants of plant growth and productivity. Amongst the plant families, leguminous plants can achieve a nitrogen-fixing symbiosis with soil bacteria of the family Rhizobiaceae to reduce atmospheric nitrogen (N_2_) to ammonia [3]. The ability to reduce N_2_ is restricted to bacteria and archaea which produce the enzyme nitrogenase. Legumes are an economically important plant family, for their contribution to animal and human nutrition on one hand, and for their ecosystemic services in cropping systems on the other hand, by participating to nitrogen enrichment of soils and thereby to a reduced use of nitrogen fertilizers. The study of these symbioses is therefore a major challenge to promote a more environmentally-friendly agriculture.

The nitrogen-fixing symbiosis (NFS) between rhizobia bacteria and legumes leads to the formation of new root organs, called nodules [4,5]. The development of the nodule requires many crucial steps to achieve the fixation of atmospheric nitrogen. The first step is the cross recognition between bacteria and the plant partner. This recognition involves the nodulation (Nod) factors produced by the bacteria that play a major role in the symbiotic specificity between the two partners. In parallel to bacterial recognition, Nod factors promote the development of a new meristem in the plant root that leads to the establishment of the root nodule. Subsequently, the formation of infection threads allows the transport of bacteria from the surface of the root to the plant cells that will host bacteria, in an endosymbiotic way. The accommodation of numerous bacteria inside plant cells and nitrogen fixation requirements involve modifications of the cellular structure and physiology of both partners for maintaining the symbiotic interaction. These modifications are achieved through differentiation of the plant cells which includes cell enlargement, DNA endoreduplication, and significant reprogramming of cellular structure and metabolism [6]. Cellular and biochemical changes are also observed during the differentiation of bacteria into N_2_-fixing bacteroids. Amongst them, a high aerobic metabolism provides ATP and reductants necessary to sustain nitrogenase activity, whereas the nitrogen-fixing enzyme is irreversibly inactivated by oxygen. Thus nitrogen-fixation efficiency depends on oxygen protective mechanisms involving the formation of an oxygen barrier cell layer around the infected cells and the production of a symbiotic hemoglobin, called leghemoglobin. This later is involved in the protection of nitrogenase from denaturation, and in the supply of ample amount of oxygen to bacteria for respiration. The supply of energy from the plant to nitrogen-fixing bacteroids, and the export of ammonia from the bacteroids to the roots also require major metabolic adaptations in the nodules. In conclusion, the nodule functioning depends on a strict regulation of the development and the metabolism of plant and bacteria cells.

**Figure 1:**
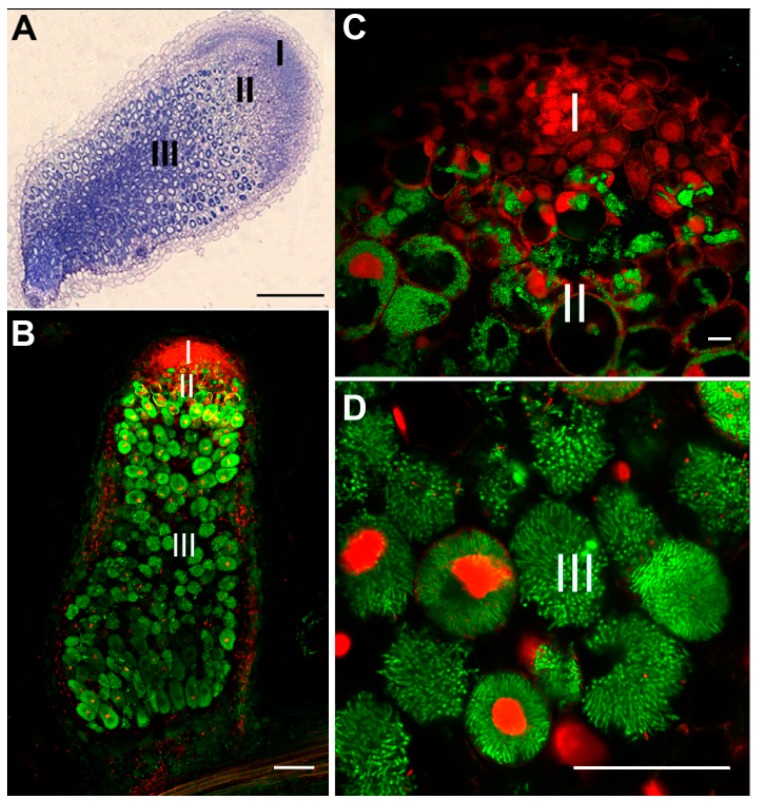
The indeterminate root nodule structure in *Medicago truncatula*. (**A**) Longitudinal section of indeterminate nodule 3 weeks post infection (wpi) with the apical meristem (I), the infection zone (II), and the nitrogen-fixing zone (III). (**B**–**D**) Longitudinal section of wild-type nodules 3 wpi analyzed by confocal microscopy with *S. meliloti* DNA stained with SYTO9 (green) and plant nuclei stained with propidium iodide (red) [8]. (**C**) The size of plant cells and of plant cell nuclei increases during cellular differentiation and intracellular bacterial infection occurs in zone II. (**D**) The nitrogen-fixing cells in zone III are fully packed with numerous elongated endosymbiotic bacteria called symbiosomes. Bars: (**A**) 200 µm; (**B**) 100 µm; (**C**) 10 µm; (**D**) 50 µm.

The nodules are considered as “indeterminate” or “determinate” according to their mode of development [7]. In the determinate nodules, such as those of soybean (*Glycine max*), the nodular meristems are transiently active. This results in spherical nodules, containing cells with a similar developmental state to each other. In the indeterminate nodules, such as those formed by pea (*Pisum sativum*), alfalfa (*Medicago sativa*) or barrel medic (*M. truncatula*), the meristems persist throughout the plant’s life, giving an elongated nodule. Consequently, the functional nodule presents three zones: (I) the meristematic zone, (II) the infection zone, and (III) the N_2_-fixing zone (Figure 1A,B). At later stage, there is a rupture in the symbiotic interaction, which occurs in the senescence zone (zone IV).

As mentioned above, the meristematic cells of the nodule destined to house the rhizobia undergo several DNA endoreduplication cycles. The endoreduplication (up to 64 C) is accompanied by an expansion (up to 80 times) of the infected cells (Figure 1C) [9,10]. These transformations are associated with metabolic changes allowing the bacteria reception and the assimilation of reduced nitrogen. Bacteroid differentiation depends on the host plant [11]. In some legumes such as soybean, the bacteroid morphology is little affected in comparison to the free-living bacteria. In contrast, in other legumes, such as faba bean (*Vicia faba*), pea or the *Medicago* genus, bacteroids present an extreme morphological change with an elongated phenotype (5 to 10 times longer than the free-living cells) (Figure 1D). This change is coupled with endoreduplication of the bacterial genome, and irreversible terminal differentiation, preventing subsequent bacterial multiplication. Transcriptomic analyses of the host plants, inducing (*M. truncatula*) or not (*Lotus japonicum*) the terminal bacterial differentiation, have allowed the identification of plant factors involved in this process. These factors, called Nodule Specific Cysteine Rich (NCR) peptides, are defensin-like peptides specifically expressed in the nodule [12]. This family of peptides has been extensively described in *M. truncatula*, and several homologs have been found in *M. sativa* and *P. sativum* [6]. The NCR peptides produced by the host plant are targeted to the bacteroids through the secretory pathway. In vitro treatment of *S. meliloti* culture with certain NCR peptides induces some aspects of terminal differentiation such as bacterial membrane permeabilization, cell division inhibition, genome endoreduplication, and bacterial elongation. In addition, mutants of *M. truncatula* deficient in two NCR peptides, NCR169 and NCR211, develop non-functional nodules [13,14].

Regulation of the cellular redox state represents a major regulatory component of the nitrogen-fixing symbiosis. During the last twenty years, analysis of numerous redox components of the nodules has shown their specific involvement in the functioning of the root nodule. Amongst them, some NADPH oxidases, which are involved in the production of reactive oxygen species (ROS), have been shown to regulate the symbiotic interaction throughout the lifetime of the nodule from its installation to its senescence [15]. Similarly, enzymes implicated in the steady state of nitric oxide (NO), a growth and metabolic regulator in plants, control nodule development and functioning [16]. Antioxidant components of the cells involved in the regulation of the cellular redox state also participate in the nodule development [17,18]. In this review, we will present an overview of the work performed on the glutaredoxin and thioredoxin systems, which regulate the redox state of the proteins, in the nitrogen-fixing symbiosis in both symbiotic partners.

## 2. The Glutaredoxin and Thioredoxin Systems of Plant Partner

### 2.1. The Glutaredoxin System

Glutaredoxins (Grxs) are small redox enzymes of approximately one hundred amino-acid residues that use glutathione (GSH) as a reducer, that is maintained in a reduced state by glutathione reductase (GR) and NADPH. The GSH synthesis has been extensively studied in leguminous plants [17]. In legumes, the structural homolog, homoglutathione (hGSH; γGlu-Cys-βAla), may partially or completely replace GSH [19,20,21]. Both compounds can be found at concentrations of 0.5–1.5 mM in nodules [22], similar to the estimated levels of 1–3 mM GSH and 0.4–0.8 mM hGSH in the chloroplast stroma [23] or in the cytosol [24]. However, the (h)GSH content is much higher in nodules than in roots due to the structural modifications of nodule cells with an increased cytosol volume compared to root cells (see Figure 1). (h)GSH synthesis derives from sulfur (S) metabolism which has been studied in N_2_ fixing nodules of *L. japonicus* [25]. The high adenosine 5′-phosphosulfate reductase activity, the strong S-flux into cysteine and derivatives, and the up-regulation of the expression of several rhizobial and plant genes involved in S-assimilation showed the important function of nodules in S-assimilation [25]. Moreover, the higher thiol content observed in roots and leaves of N_2_-fixing plants in comparison to uninoculated plants could not be attributed to local biosynthesis, showing that nodules are an important site for production of reduced S for the plants [25]. The S-metabolism of nodules is reduced in plants nodulated by mutant rhizobia unable to reduce N_2_ indicating a strong interdependency between N_2_-fixation and S-assimilation [25,26]. Sulfate transport is also modified in the nodule. Nodule-specific sulfate transporters have been identified [27]. Some of them are located on the peribacteroid membrane and allow the transport of inorganic sulfur to the bacteroid [28,29]. In soybean, this transport has been shown to be crucial for nitrogen-fixing efficiency. We have analyzed the transcriptome of *M. truncatula* using sulfate transporter as a key word in the symbimics website (https://iant.toulouse.inra.fr/symbimics/), which allows to compare the expression of genes in roots and nodules and to define the level of gene expression in the different nodule zones (Table 1). In *M. truncatula*, 22 putative sulfate transporter genes were identified. The expression of some of them (*Medtr3g087730*, *Medtr5g061860*, *Medtr6g086170*, *Mt0062_10115*) were significantly upregulated in nodules compared to roots. Transcriptomic analyses in the *M. truncatula* Gene Expression Atlas (https://mtgea.noble.org/v3/) showed that *Medtr5g061860* and *Medtr6g086170* expression were correlated with the nitrogen fixation efficiency as treatment of plants with nitrate, which reduces the nodule nitrogen fixation, led to a reduction of less than 20% of the expression of these genes as compared to control nodules.

The synthesis of GSH in plants and other organisms is accomplished in two sequential reactions catalyzed by γ-glutamylcysteine synthetase (γECS) and glutathione synthetase (GSHS), both showing a strict requirement for ATP and Mg^2+^ [23]. In legumes, the synthesis of hGSH is also carried out in two steps, involving the same γECS enzyme and a specific homoglutathione synthetase (hGSHS), which exhibits a much higher affinity for β-alanine than for glycine [19,20,30,31]. Site-directed mutagenesis of soybean and *M. truncatula* hGSHS has conclusively shown that two contiguous amino acid residues in the active site (Leu-487 and Pro-488, positions that are Ala in GSHS) mainly determine the substrate preference for β-alanine over glycine [20,32]. The *GSHS* and *hGSHS* genes share high homology (~70% amino acid identity) and are located in tandem on the same chromosome in the model plant legumes *M. truncatula* [20] and *L. japonicus* [21]. These findings are consistent with the hypothesis that the *hGSHS* gene derives from the *GSHS* gene by a duplication event occurring after the divergence between the Fabales, Solanales, and Brassicales [20]. Despite this close relationship, the two genes are differentially regulated in plant organs. This can be exemplified with studies performed on the two model legumes. Thus, *M. truncatula* produces exclusively GSH in the leaves and both GSH and hGSH in the roots and nodules, whereas *L. japonicus* produces almost exclusively hGSH in the roots and leaves, but more GSH than hGSH in the nodules. In legumes, the thiol contents are positively correlated with the GSHS and hGSHS activities and in general with their mRNA levels [21,22,23,24,25,26,27,28,29,30,31,32,33].

The concentration of (h)GSH and the N_2_-fixing activity in nodules are positively correlated during nodule development [34]. The two parameters decline with aging [35,36] as well as during stress-induced senescence [37,38,39,40,41]. These findings suggest that (h)GSH is important for nodule activity, a hypothesis that was tested by modulating the nodule content of (h)GSH using pharmacological and genetic approaches. The application of buthionine sulfoximine (a specific inhibitor of γECS) or the expression of (h)GSHS in antisense orientation caused depletion of (h)GSH in *M. truncatula* roots [42]. The (h)GSH synthesis deficiency in roots decreased substantially the number of nascent nodules and the expression of some early nodulin genes [42]. These results, along with the proposed role of GSH in meristem formation in *Arabidopsis thaliana* [43,44,45], suggest that (h)GSH is required for the initiation and maintenance of the nodule meristem. The transcriptomic analysis of (h)GSH-depleted plants during early nodulation revealed downregulation of genes implicated in meristem formation and upregulation of salicylic acid-related genes after infection with *S. meliloti* [46]. The enhanced expression of defense-related genes provides a partial explanation for the negative effects of (h)GSH depletion on the symbiosis. The role of (h)GSH was also analyzed in the nitrogen-fixing zone. Downregulation of the γECS gene by RNA interference using the nodule nitrogen-fixing zone-specific NCR001 promoter resulted in significantly lower biological nitrogen fixation (BNF) associated with a significant reduction in the expression of nodule specific genes. This lower (h)GSH content was correlated with a reduction in the nodule size. Conversely, γECS overexpression using the same promoter resulted in an elevated GSH content associated with increased BNF and significantly higher expression of the sucrose synthase-1 and leghemoglobin genes. Taken together, these data show that the plant (h)GSH content of the nodule nitrogen-fixing zone modulates the efficiency of the BNF process, demonstrating their important role in the regulation of this process [47]. All these data show the importance of sulfur metabolism and more particularly of (h)GSH in the development and functioning of nodules.

Amongst the multiple roles of (h)GSH, these thiols serve as reducing power for Grxs. Numerous Grxs are present in plants. To date, no physiological analysis was performed to investigate the importance of Grxs in the nitrogen-fixing symbiosis. *M. truncatula* genome analysis using BLAST and publication data mining [48,49] allowed us to find thirty-six genes encoding putative Grxs of class I, II, and III (Table 2). Gene expression analysis in roots and nodules did not allow us to find class I and class II Grxs significantly upregulated in nodules compared to roots. In contrast, two Class III Grxs (*Medtr2g014760*, *Medtr1g088910*) are upregulated in nodules compared to roots suggesting that they play a significant role in nodule development or functioning. Nevertheless, three class III Grxs are also significantly downregulated in nodules compared to roots and multiple Class III Grxs are not expressed in roots and nodules. Taken together, these results showing the significant modification of the expression of multiple Grx genes suggest that redox regulation of nodule metabolism is extensively modified compared to roots. 

### 2.2. The Thioredoxin System

The other biochemical system involved in the thiol-dependent redox regulation of enzyme activity is the thioredoxin system. Thioredoxins (Trxs) are small proteins similar to Grxs that reduce disulfide bounds. Oxidized Trxs are in turn re-reduced by NADP-dependent Trx reductases (TR) and NADPH or ferredoxin in plastids. Nonetheless, a few members of the Trx family use, as Grxs, glutathione as a reducer [51,52]. Trxs are able to reduce directly some of basic metabolic enzymes such as ribonucleotide reductase, and enzymes involved in the antioxidant systems such as peroxiredoxins (Prx), glutathione peroxidase (Gpx), and methionine sulfoxide reductase (MSR). In plant tissues, several groups of Trxs have been identified. The Trxs f, m, x, y, and z are localized in the plastids, Trxs o are addressed to the mitochondria and the Trxs h mainly accumulate in the cytoplasm [53]. Cytosolic Trxs h can also be transferred in nucleus in cells suffering oxidative stress [54]. Nucleoredoxins were also described as other redoxins located in the nucleus [55]. In legumes, the Trx family has been analyzed in detail in *M. truncatula* [52,56] and *L. japonicus* [57]. The analysis of *Trx* expression in *L. japonicus* showed that there is a differential expression pattern of the different isoforms in leaves, root, and nodules. However, no isoform seems to be significantly more expressed in nodules than in roots and leaves. In soybean, a Trx h expressed in infected cells of mature nodules is able to protect a yeast Trx mutant against hydrogen peroxide (H_2_O_2_) [58]. This Trx is crucial for nodule development and functioning as RNAi-mediated repression of the Trx gene severely impaired nodule development [58]. Nodulin-35, a subunit of uricase, was found to be a target of this thioredoxin suggesting a novel role of Trx in the regulation of enzyme activities involved in nodule nitrogen fixation [59]. In addition to all the classical types of Trxs found in plants, *M. truncatula* contains a novel type of Trxs, called Trxs s, comprising four isoforms which are associated with symbiosis [56,60]. No orthologs were found in *A. thaliana*, *L. japonicus* or soybean suggesting that the Trxs s isoforms could be unique to certain legume species. *Trx s1* and *s3*, are induced in the nodule infection zone where bacterial differentiation occurs. Trx s1 is targeted to the symbiosomes, the N_2_-fixing organelles. Trx s1 interacted with NCR247 and NCR335 and increased the cytotoxic effect of NCR335 in *S. meliloti*. *Trx s1* silencing impairs bacteroid endoreduplication and enlargement, two features of terminal bacteroid differentiation, and the ectopic expression of *Trx s1* in *S. meliloti* partially complements the silencing phenotype. Thus, Trx s1 is targeted to the bacterial endosymbiont where it controls bacteroid terminal differentiation [60]. 

Gpxs and Prxs are also present in root nodules. In plants, most Gpxs reduce hydroperoxides using Trxs and TR, instead of GSH and GR, as a reducing system [61]. This is also true for Gpx1 in *M. truncatula* [62]. Based on genome analysis, six Gpxs were reported in *L. japonicus* [63]. Except the *LjGpx4*, the other isoforms were expressed in nodules with a higher level for *LjGpx1* and *LjGpx3* [63,64]. The two Gpx were Trx-dependent phospholipid hydroperoxidases and were upregulated in response to NO for LjGpx1 and in response to cytokinine and the ethylene precursor ACC for LjGpx3 [64]. Both genes were highly expressed in the nodule zone containing the bacteria, and the *LjGpx3* mRNA was also detected in the cortex and vascular bundles. Immunogold localization of Gpx allowed to localize LjGpx1 in plastids and nuclei and LjGpx3 in the cytosol and the endoplasmic reticulum [64]. Based on yeast complementation experiments, both enzymes protect against oxidative stress, salt stress, and membrane damage suggesting that both LjGpxs perform major antioxidative functions in nodules, preventing lipid peroxidation and other oxidative processes at different subcellular sites of vascular and infected cells [64]. 

There are four types of Prxs in plants (1-CysPrx, 2-CysPrx, PrxII, and PrxQ). Based on genome analysis, seven Prxs were reported in *L. japonicus* [63]. Eight transcripts were detected: *Lj1CPrx*, *LjPrxQ1a*, and *LjPrxQ1b* which derive from the gene *LjPrxQ1a* with an alternative splicing, and *Lj2CPrxA*, *Lj2CPrxB*, *LjPrxIIB*, *LjPrxIIE*, and *Lj1CPrxIIF* [63]. The expression profiles in the different plant tissues did not allow the detection of a Prx isoform which would be more expressed in the nodules. Nevertheless, reduction of *PrxIIB* and *PrxIIF* expression levels were associated to the nodule senescence process in bean nodules [65]. In contrast, whereas the level of PrxIIF protein remains constant in senescent nodules, the level of PrxIIB decreases in senescent nodules [65]. Similarly, the decrease of the putative PrxIIA content and a constant level of the mitochondrial PrxIIF protein were observed in senescent nodules compared to mature nodules [66]. Trx also serve as electron donors for MSRs that repair oxidized proteins. To our knowledge, no experiment was performed to analyze the roles of MSRs in root nodules. We have analyzed the transcriptome of *M. truncatula* searching for methionine sulfoxide in the symbimics website and BLAST sequence alignment program to validate the putative identity of the sequences. The comparison of gene expression in roots and nodules, and in the different nodule zones allowed the detection of a significant upregulation of *Medtr3g051460* in the infection zone and the nitrogen-fixing zone compared to the uninfected nodule zone I. Apart from this isoform, no clear difference in transcript level was observed for the seven other genes. Functional analysis of this enzymatic family awaits to be performed in root nodules. 

## 3. The Glutaredoxin and Thioredoxin Systems of Bacterial Partner

Rhizobial genomes contain the genes of Grx and Trx systems (http://genome.annotation.jp/rhizobase). We summarize recent data on these systems emphasizing how they contribute to the efficiency of nitrogen-fixing symbiotic interaction. 

### 3.1. The Gutaredoxin System

In most bacteria, the glutaredoxin system consists of GR, which catalyzes the NADPH-driven reduction of glutathione disulfide (GSSG) to GSH, which in turn reduces Grx. The two steps of GSH biosynthesis are catalyzed by γECS and GSHS, encoded by the *gshA* and *gshB* genes, respectively. The GSH recycling from GSSG is performed by a GR encoded by the *gor* gene. Studies of rhizobial mutants affected in GSH metabolism demonstrate the central role of GSH pool in free-living cells and in planta. In all cases, *gshB* inactivation alters the fitness of free-living bacteria, and *gshB* mutants develop poorly effective symbiosis with their plant partners. For example, the growth of a *S. meliloti gshB* mutant is altered in minimal medium whereas a *gshA* mutant does not grow under the same conditions, showing that GSH is essential and can be partially replaced by γ-glytamyl-cysteine [67]. The two mutants experience oxidative stress as both exhibit higher catalase activity, a biochemical marker of oxidative stress, when compared with the wild-type strain. *M. sativa* plants inoculated with the *gshA* mutant did not produce nodules, while *gshB* inactivation triggered a delayed nodulation phenotype and the development of abnormal, early senescing nodules associated with 75% reduction in the nitrogen-fixation capacity of bacteroids. A *gshB* mutant of *Rhizobium tropici* has a reduced ability to compete against the wild-type strain for nodule occupancy on common bean, while a *Rhizobium etli gshB* mutant has a delayed nodulation phenotype when inoculated onto bean [68,69]. Plants infected by either one of the other *gshB* mutant develop ineffective nitrogen-fixing nodules with obvious signs of early senescence. Nodule phenotype is associated with enhanced levels of superoxide anion in the case of *R. tropici* infection, showing that GSH-deficient bacteroids face an environmental oxidative stress [68]. In the same way, a *gshA* mutant of *Bradyrhizobium japonicum* gives rise to nodules with a strong nitrogen-fixation deficiency during interaction with soybean [70]. There are, however, variations in GSH requirement among rhizobial species since another *Bradyrhizobium*-legume interaction develops effective nodules independently of the bacterial GSH pool. The *gshA* mutant of *Bradyrhizobium* sp. SEMIA 6144 indeed induced functional nodules with peanut (*Arachis hypogaea* L.), even though GSH depletion affects nodule occupancy capacity and growth of the free-living bacterium in normal and stressful conditions [70]. Overall, the homeostasis (both level and redox status) of GSH in bacterial cells is important for nodule development, and this is also exemplified by the symbiotic deficiency of *S. meliloti gor* mutant [71]. The lack of GR in *gor* mutants causes a decrease in the GSH/GSSG ratio, triggering oxidative stress with an increased expression of catalase genes, and an enhanced sensitivity to oxidants. In planta, the *gor* mutant is affected in its ability to compete for nodule occupancy and displays a reduced nitrogen-fixing phenotype [71]. Altogether, these different studies highlight the major role of rhizobial GSH in regulating the intracellular redox environment and protecting cells against ROS. 

Besides its role in redox balance, the GSH pool in nodules might also be crucial in regulating metabolic pathways. The *R. etli gshB* and *gor* mutants were shown to be affected in Gln uptake in free-living bacteria [69]. Similarly, a *gshB* mutant of *Rhizobium leguminosarum* is impaired in symbiosis with *P. sativum* and presents a defect in the uptake of several carbon source compounds in free-living bacteria [72]. 

Glutathione is involved in the maintenance of cellular redox homeostasis in particular as a reductant for Grxs. The function of bacterial Grxs during rhizobium–legume symbiosis has been investigated in *S. meliloti*. The genome of this bacterium encodes three Grxs, the dithiol SmGrx1 (CGYC redox active site), the monothiol SmGrx2 (CGFS redox active site), and the atypical SmGrx3 which carries two domains, an N-terminal Grx domain with a CPYG active site and a C-terminal domain with a methylamine utilization protein (MauE) motif. Both SmGrx1 and SmGrx2 orthologs are ubiquitously present in bacteria while SmGrx3 orthologs are found only in cyanobacteria and some proteobacteria [73]. Biochemical and genetic analyses established that the three proteins have distinct properties [73]. SmGrx1 was shown to play a key role in protein deglutathionylation: on one hand SmGrx1 recombinant protein displayed an efficient degluthationylation activity, on the other *Smgrx1* inactivation in free-living bacteria led to a higher level of glutathionylated proteins. The *Smgrx1* deficient mutant undergoes a severe growth defect under non-stress conditions and an increased sensitivity to H_2_O_2_ treatment. During the interaction with *M. truncatula* the *Smgrx1* mutant induces abortive nodules, containing bacteria unable to differentiate into bacteroids following release inside plant cells. This original symbiotic phenotype suggests that the control of protein and redox homeostasis by Grx1-mediated protein deglutathionylation is crucial for bacteroid differentiation. 

Data obtained with SmGrx2 provide the first demonstration of Grx involvement in bacterial iron metabolism [73]. *Smgrx2* inactivation in free-living bacteria results in the decreased activity of Fe–S cluster containing enzymes, suggesting that SmGrx2 participates to Fe–S cluster assembly machinery. A deregulation of RirA (Rhizobial iron regulator)-dependent genes, and an increase of the total intracellular iron content, was also observed in the *Smgrx2* mutant. During the interaction between *S. meliloti* and *M. truncatula*, *Smgrx2* inactivation affects nodulation efficiency and the nitrogen-fixation capacity of bacteroids; *Smgrx2* bacteroids are fully differentiated, in contrast to those of *Smgrx1*. The nitrogen-fixation deficiency of mutant bacteroids could result from a direct effect on nitrogenase which contains many Fe–S clusters. Indeed, the nitrogen-fixing enzyme consists of two Fe–S cluster-containing proteins, the dimeric Fe protein that serves as the electron donor for N_2_ reduction and as the site of ATP hydrolysis, and the heterotetrameric MoFe protein where substrates are reduced. The Fe protein contains a Fe–S cluster while the MoFe protein contains two unique metal clusters, the [8Fe:7S] P-cluster and the FeMo cofactor described as a [Mo:7Fe:9S]:C-homocitrate entity [74]. Consistently, a mutant in *sufT*, involved in Fe–S cluster metabolism, also has a lowered nitrogen fixation capacity [75]. 

Concerning SmGrx3, the same approaches used to analyze SmGrx1 and SmGrx2 function were performed. Whereas a SmGrx3 recombinant protein presents a low degluthationylation activity, a *Smgrx3* mutant did not display defective phenotype in the free-living and symbiotic states. The biological function of SmGrx3 still remains to be elucidated.

In conclusion, SmGrx1 and smGrx2 play distinct, critical roles in the control of *S. meliloti* physiology. The growth and symbiotic defects of *grx* mutants also indicate that Grx and Trx systems are not functionally redundant in *S. meliloti*, in contrary to the thiol-redox systems of *E. coli* [76]. The question arises as to whether these properties can be generalized to other rhizobial Grxs and deserves more studies. 

### 3.2. The Thioredoxin System

The thioredoxin system consists of NADPH, the flavoprotein Trx reductase (TR), and Trxs. A very limited number of studies have investigated the role of thioredoxin system in rhizobia. Trx-like proteins were initially described as playing an important role in symbiosis. In *S. meliloti* CE52G, inactivation of a *trx*-like gene involved in melanine production increased the sensitivity of free-living bacteria to paraquat-induced stress and affected the nitrogen fixation capacity of bacteroids [77]. In *R. leguminosarum*, a mutant deficient in the Trx-like TlpA, involved in cytochrome c biogenesis, was unable to form nitrogen-fixing nodule [78]. TlpA was recently shown to act as a reductant for the copper metallochaperone ScoI and cytochrome oxidase subunit II CoxB [79]. 

In *S. meliloti* as in *E. coli*, the canonical Trx system contains two Trxs, TrxA and the product of *SMc03801* (TrxC in *E. coli*), and one TR (TrxB). Recent results showed that TrxB recombinant protein efficiently reduces Trx s1, a host-plant thioredoxin specifically addressed to the microsymbiont, which is able to reduce NCR and is involved in bacteroid differentiation [60]. These data suggest that TrxB is implicated in the redox regulation of differentiation by reactivating Trx s1 but further studies are required to characterize the physiological role of TrxB during symbiosis. 

### 3.3. Transcriptional Regulation of Trx and Grx Systems in S. meliloti

Various gene expression studies underline the importance of rhizobial Trx and Grx systems during symbiosis; we will focus on *S. meliloti* for which most of the results were obtained.

A high expression level of Trx/Grx component genes was observed in bacteroids from different zones of the *M. truncatula* nodules ([50]; Table 3). Some of these genes belong to stress response regulons, markedly required for *S. meliloti* survival in host cells. 

Regulation of the *S. meliloti* GSH metabolic pathway involves the activity of LsrB, a transcriptional regulator required for efficient alfalfa nodulation [80]. LsrB belongs to the LysR family of bacterial transcriptional regulators including the oxidative stress regulator OxyR [81]. An *lsrB* deletion mutant has a reduced pool of GSH, and LsrB inactivation accordingly results in the decreased expression of genes involved in GSH metabolism (*gshA*, *gshB*, *gor*) both in free-living and in planta [82,83]. The regulator was shown to directly activate the expression of *gshA*, and to respond to cellular redox changes via the three reactive cysteines in the substrate-binding domain [83]. LsrB also positively regulates the expression of genes involved in lipopolysaccharide biosynthesis [84]. Nodules induced by mutants defective in LsrB undergo premature senescence coupled to impaired bacteroid differentiation and ROS accumulation, which could be partly due to GSH deficiency [83]. Several genes of the Trx/Grx systems belong to the RpoH1 regulon. RpoH1 is one of the 14 alternative sigma factors encoded in the *S. meliloti* genome. The presence of multiple RpoHs in *S. meliloti* and other alpha proteobacteria is correlated with a diverse lifestyle. RpoH1 regulates gene expression in response to acidic pH stress [85,86], heat shock, and stationary phase [87], and was also involved in maintaining the redox status of the cell challenged with H_2_O_2_ [88]. A *rpoH1* mutant is capable of eliciting the formation of nodules on alfalfa plants, but shows poor survival after its release in plant cells and barely fixes N_2_ [89]. In addition to environmental stresses encountered both in free-living state and in planta, the *S. meliloti* microsymbiont is challenged with hundreds of peptides secreted by the host-plant, and largely involved in controlling bacterial populations during nodule development and functioning [90]. Transcriptome analyses of cultures challenged with two cationic NCR peptides exhibiting antimicrobial activities, NCR247 and NCR335, showed upregulation of genes involved in stress adaptation such as *Smgrx1* and *trxA* [91], see Table 3. This effect might be mediated via RpoH1, as the *rpoH1* gene itself was induced by NCR treatment [91].

Other, still unknown transcription factors and signals are likely also to be involved in the regulation of Grx/Trx systems, and other regulatory mechanisms as well. For example, the expression of *gshB* and Sm*grx2* is very low in zone III whereas the activity of GSHS and SmGrx2 is required in this zone, indicating that post-transcriptional regulation mechanism(s) could play a significant role. 

Some well-known target proteins of Trx or Grx have a high expression level inside the nodules and might contribute to their optimal development (Table 3). Ribonucleotide reductase (RNR) plays a central role in DNA replication and repair by catalyzing production of deoxyribonucleotides from the corresponding ribonucleotides. Both Trx and Grx were identified as being dithiol electron donors for the *E. coli* RNR [92]. There are three major classes of RNRs based on the metallocofactors necessary for nucleotide reduction. *S. meliloti* requires a cobalamin-dependent class II RNR for symbiosis with *M. sativa* [93]. This RNR is most likely involved in DNA synthesis during bacteroid differentiation, when cells undergo endoreduplication, and later in DNA repair within differentiated bacteroids. The *nrdJ* gene encoding RNR has a maximal expression in zone III, suggesting that the level of RNR synthesis and DNA repair mechanisms are tightly linked in bacteroids. Trxs are also involved in protein repair by providing the electrons to peptide methionine sulfoxide reductases (MsrA/MsrB), which catalyze the reduction of methionine sulfoxides (S- and R-MetSO diastereosisomers respectively) back to methionine [94]. Three *msrA* and three *msrB* genes are present in the genome of *S. meliloti.* The highest level of *msrA/msrB* expression in nodules is observed in the differentiation and nitrogen fixation zones, a feature probably correlated to an increased methionine oxidation once bacteroid differentiation has begun. Most *msrA/msrB* genes are controlled by RpoH1, which underlines the coordinated expression of genes encoding components of Trx system and their targets.

*S. meliloti* encodes thiol-base peroxidases of distinct families, one typical 1-Cys peroxiredoxin (product of *SMb20964*), and two organic hydroperoxide resistance thiol peroxidase paralogs from the OsmC/Ohr family [95]. Whereas Ohr proteins used a lipoylated protein as reductant, the bacterial 1-Cys or 2-Cys peroxiredoxins can use Trx or GSH as reductants to support small alkyl peroxide or H_2_O_2_ reductase activity [96,97]. The *S. meliloti* 1-Cys peroxiredoxin is upregulated in cultures challenged with H_2_O_2_ via an OxyR-dependent mechanism [88]. The corresponding protein has been identified in nodules [98] and transcripts were detected in bacteroids mostly in zone III [50], suggesting a role in nodule functioning. In *R. etli*, the typical 2-Cys peroxiredoxin PrxS uses the thioredoxin system for H_2_O_2_ reductase activity. The *R. etli* double mutant *prxS*-*katG*, deficient for both peroxidoxin and catalase-dependent H_2_O_2_ reduction, induced nodules with reduced nitrogen-fixation capability [99].

In conclusion, these data suggest the existence of a complex oxidative stress response network involving Grx and Trx to control protein redox state, and which allow bacteria to adapt to the host cell environment. 

## 4. Conclusions

**Figure 2:**
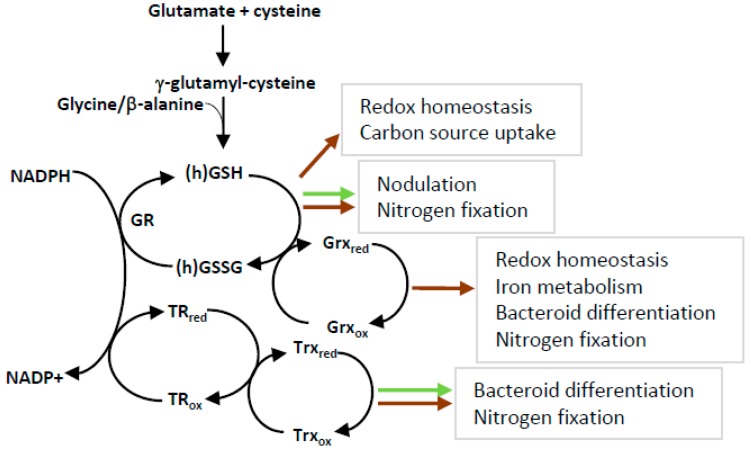
An overview of the physiological importance of Trx and Grx networks in rhizobium–legume symbiosis. Redox networks of glutaredoxin and thioredoxin systems in the two symbiotic partners is shown. The roles of (h)GSH, Grxs, and Trxs (grey squares) are indicated for bacteria (brown arrow) and for plants (green arrow). See text for details.

During the last years, many advances have been made in the characterization of redox regulatory systems and their roles in the two partners of nitrogen-fixing symbiosis (Figure 2). The development of genomic and transcriptomic analyses allowed to better characterize the gene families involved in redox metabolism and to define their expression regulation. A new research track was also opened with the redox regulation of the cross-talk between plant and bacteria, exemplified by Trx s1. However, these advances are revealing the complexity of the regulatory mechanisms and an increased number of key regulatory actors, as illustrated by the amazing high number of Grxs and Trxs in the plant partner. Characterizing the most promising candidates represents an important task both at the scientific level and in terms of work amount. Moreover, many lines of research remain to be opened. One of them is to assemble the different pieces of the “redox puzzle”, taking into account the redox post-transcriptional regulation signals including oxidation, nitrosylation, and glutathionylation. In this perspective, the development of redox proteomics and laser microdissection will allow the large-scale identification of proteins that are modified in response to specific stimuli in specific cell types. Another crucial task would be the use of this huge amount of knowledge to improve the resistance of nitrogen fixation efficiency to abiotic stresses. Intensive farming leads to a significant increase in surfaces affected by drought or salinity, which particularly impairs nitrogen fixation. In the context of sustainable agriculture, the use of redox components as markers for symbiotic efficiency or as genetic material to improve plant breeding or bacterial inoculum is of crucial importance. 

## Figures and Tables

**Table 1:** Expression of plant sulfate transporters in *M. truncatula* nodules. Gene accession numbers are indicated in the table. Gene annotation is based on candidate orthologues and interprodomain signature. The different columns correspond to root and nodule whole organ analysis (Root and Nodule) and to the nodule zones : meristematic zone (I), distal infection zone (IId), proximal infection zone (IIp), infection/fixation interzone (IZ II-III) and nitrogen-fixation zone (III). The numbers in the different columns correspond to Total Reads ribominus. All RNA-seq read values were normalized [50]. The total reads are reported from the symbimics bioinformatics website. The full organs are nitrogen starved Roots and 10 days old nodules. The red and blue colours correspond respectively to higher and lower significant differences between the organs (roots and nodules) and between the different nodule zones. The statistical differences are reported from the symbimics bioinformatics website.

Gene Name	Root	Nodule	I	IId	IIp	IZ II-III	III
Sulfate transporter							
*Medtr7g095430*	1307	1297	7	2	4	19	9
*Medtr4g084620*	298	176	13	9	43	44	29
*Mt0062_10115*	54	548	30	154	289	235	361
*Medtr4g011970*	633	1052	307	1721	3064	1094	1406
*Medtr3g087730*	4	876	118	530	1088	323	92
*Medtr5g061860*	87	1449	4	14	22	1313	1742
*Medtr6g086170*	45	48,527	14	291	566	6898	12,537
*Medtr3g073730*	220	20	2	4	1	1	0
*Medtr5g061880*	200	43	15	14	2	3	3
*Medtr2g102243*	322	193	19	18	1	1	0
*Medtr2g008470*	1406	253	1	1	1	7	8
*Medtr3g087740*	2150	854	19	17	16	4	13
*Mt0050_00072*	0	0	0	0	0	0	0
*Medtr4g084640*	0	0	0	0	0	0	0
*Mt0008_01149*	0	0	0	0	0	0	0
*Mt0008_11083*	0	0	0	0	0	0	0
*Medtr4g063825*	0	6	1	0	0	0	0
*Mt0006_10002*	1	0	2	3	0	0	0
*Medtr2g082610*	3	5	1	2	0	0	0
*Medtr3g073780*	9	1	0	0	0	0	1
*Medtr1g071530*	345	184	2	2	0	1	1
*Medtr7g022870*	464	449	48	52	17	20	39

**Table 2:** Expression of glutaredoxins in *M. truncatula* nodules. Gene accession numbers are indicated in the table. Gene annotation is based on candidate orthologues and interprodomain signature. The different columns correspond to root and nodule whole organ analysis (Root and Nodule) and to the nodule zones: meristematic zone (I), distal infection zone (IId), proximal infection zone (IIp), infection/fixation interzone (IZ II-III) and nitrogen-fixation zone (III). The numbers in the different columns correspond to Total Reads ribominus. All RNA-seq read values were normalized [50]. The total reads are reported from the symbimics bioinformatics website. The full organs are nitrogen starved Roots and 10 days old nodules. The red and blue colours correspond respectively to higher and lower significant differences between the organs (roots and nodules) and between the different nodule zones. The statistical differences are reported from the symbimics bioinformatics website.

Gene name	Putative redox site	Root	Nodule	I	IId	IIp	IZ II-III	III
Glutaredoxins								
**Class I**								
*Medtr7g035245*	YCPFC	2612	2354	665	163	156	238	167
*Medtr1g069255*	WCSYC	121	153	54	41	135	109	92
*Medtr3g077560*	YCGYC	1314	366	2	1	1	2	0
*Medtr3g077570*	YCGYC	201	148	27	16	3	3	15
*Medtr2g038560*	YCPYC	1573	1200	425	682	837	509	297
*Medtr5g021090*	YCPYC	1284	1444	76	97	63	72	50
**Class II**								
*Medtr2g103130*	QCGFS	1332	1390	380	266	323	546	302
*Medtr4g079110*	GCCMS	968	1834	62	36	1	0	0
*Medtr7g079520*	QCGFS	771	640	170	143	103	107	62
*Medtr4g088905*	KCGFS	1444	1940	277	252	194	97	105
*Medtr4g016930*	LCGSF	124	218	57	66	70	65	71
**Class III**								
*Medtr7g026770*	TCCMC	13	13	21	5	0	0	0
*Medtr3g104510*	SCCMC	16	32	1	3	24	53	96
*Medtr1g088910*	SCYMC	62	260	2	0	0	0	0
*Medtr1g015890*	SCCMC	144	340	100	1437	562	164	258
*Medtr2g090755*	GCCMS	78	387	10	4	1	2	3
*Medtr2g014760*	GCCLC	71	467	33	19	8	4	10
*Medtr1g088925*	SCCLC	474	1	1	1	0	0	0
*Medtr1g088920*	LCCLC	460	3	1	0	0	0	0
*Medtr7g108200*	SCCLC	1650	308	2	1	0	0	0
*Medtr4g119030*	SCCMS	0	0	0	0	0	0	0
*Medtr2g048970*	SCCMS	0	0	0	0	0	0	0
*Medtr2g019950*	SCGMS	0	0	0	0	0	0	0
*Medtr4g119050*	SCCMS	0	0	0	0	0	0	0
*Medtr7g108250*	TCCLS	0	0	0	0	0	0	0
*Medtr7g108220*	SCYMC	0	0	0	0	0	0	0
*Medtr7g108250*	TCCLS	0	0	0	0	0	0	0
*Medtr7g022690*	SCCMC	0	0	0	0	0	0	0
*Medtr5g077550*	DCCFS	0	0	0	0	0	0	0
*Medtr1g088905*	TCCLS	0	0	1	0	0	0	0
*Mt0001_10735*	SCCMS	0	1	0	0	0	0	0
*Medtr7g022710*	SCCMC	0	0	1	0	0	0	0
*Medtr7g022550*	SCCMC	0	0	1	0	0	0	0
*Medtr7g108260*	TCPMS	2	4	0	0	0	0	0
*Medtr2g019900*	SCCMC	16	84	0	0	0	0	0
*Medtr7g108210*	SCYMC	30	30	1	0	0	0	0

**Table 3:** Expression of *S. meliloti* genes from the Grx and Trx systems in *M. truncatula* nodules and regulation in free-living bacteria. Gene accession numbers are indicated in the table. Gene annotation is based on candidate orthologs and interprodomain signature. The values corresponding to gene expression in root nodules are, from left to right, total reads from laser-capture microdissection (LCM) and their distribution in each zone (%), as reported by Roux and colleagues [50]. All RNA-seq read values were normalized. The full organs were 10-day old nodules. IZ, interzone; ZIII, zone III; FI, fraction I; FIId, distal fraction II; FIIp, proximal fraction II.

*S. meliloti* Genes	Bacterial Gene Expression in *M. truncatula* Nodules						Transcription Factors	Inducing Conditions	References
	Total reads LCM	% FI	% FIIp	% FIId	% IZ	% FIII			
**Grx system**									
*SMc00825 (gshA)*	3819	16	22	30	16	16	LsrB	GSSG	[82,83]
*SMc00419 (gshB)*	6433	8	9	24	53	6	LsrB, RpoH1		[82,87]
*SMc00154 (gor)*	4477	19	12	28	24	17	LsrB, RpoH1		[82,87]
*SMc02443 (Smgrx1)*	9123	7	6	20	16	51	RpoH1	low pH	[86]
								NCR247, NCR335	[91]
*SMc00538 (Smgrx2)*	10138	24	24	21	22	9			
*SMa0280 (Smgrx3)*	1571	17	18	22	22	21		HS	[87]
**Trx system**									
*SMc02761 (trxA)*	5519	13	10	19	21	37	RpoH1	HS	[87]
								NCR247, NCR335	[91]
*SMc03801*	2780	20	18	31	14	17	RpoH1		
*SMc01224 (trxB)*	5394	18	17	28	13	24	RpoH1	low pH, HS	[86,87]
**Grx/Trx targets**									
*SMc02885 (msrA1)*	2016	11	8	17	32	33	RpoH1	low pH, HS	[86,87]
								NCR247, NCR335	[91]
*SMc02467 (msrA2)*	1690	5	14	22	43	16			
*SMa1896 (msrA3)*	551	20	12	26	20	21	RpoH1	HS	[87]
								NCR247, NCR335	[91]
								H_2_O_2_	[88]
*SMc00117 (msrB1)*	729	24	13	19	23	20	RpoH1	HS	[87]
*SMa1894 (msrB2)*	107	0	14	17	24	45	RpoH1	HS	[87]
								NCR247, NCR335	[91]
								H_2_O_2_	[88]
*SMc01724 (msrB3)*	795	0	19	23	18	40			
*SMc01237 (nrdJ)*	33647	9	7	4	4	76			
*SMb20964*	1308	29	9	9	7	45	OxyR	H_2_O_2_	[88]

GSSG, glutathione disulfide; HS, heat shock.
